# The role of inflammatory system genes in individual differences in nonverbal intelligence

**DOI:** 10.18699/VJGB-22-22

**Published:** 2022-03

**Authors:** R.F. Enikeeva, A.V. Kazantseva, Yu.D. Davydova, R.N. Mustafin, Z.R. Takhirova, S.B. Malykh, Y.V. Kovas, E.K. Khusnutdinova

**Affiliations:** Institute of Biochemistry and Genetics – Subdivision of the Ufa Federal Research Centre of the Russian Academy of Sciences, Ufa, Russia Bashkir State University, Department of genetics and fundamental medicine, Laboratory of neurocognitive genomics, Ufa, Russia; Institute of Biochemistry and Genetics – Subdivision of the Ufa Federal Research Centre of the Russian Academy of Sciences, Ufa, Russia Bashkir State University, Department of genetics and fundamental medicine, Laboratory of neurocognitive genomics, Ufa, Russia; Institute of Biochemistry and Genetics – Subdivision of the Ufa Federal Research Centre of the Russian Academy of Sciences, Ufa, Russia Bashkir State University, Department of genetics and fundamental medicine, Laboratory of neurocognitive genomics, Ufa, Russia; Bashkir State University, Department of genetics and fundamental medicine, Laboratory of neurocognitive genomics, Ufa, Russia Bashkir State Medical University, Department of medical genetics and fundamental medicine, Ufa, Russia; Bashkir State University, Department of genetics and fundamental medicine, Laboratory of neurocognitive genomics, Ufa, Russia; Psychological Institute of the Russian Academy of Education, Moscow, Russia Lomonosov Moscow State University, Department of psychology, Moscow, Russia; Bashkir State University, Department of genetics and fundamental medicine, Laboratory of neurocognitive genomics, Ufa, Russia Goldsmiths, University of London, Department of psychology, London, United Kingdom; Institute of Biochemistry and Genetics – Subdivision of the Ufa Federal Research Centre of the Russian Academy of Sciences, Ufa, Russia Bashkir State University, Department of genetics and fundamental medicine, Laboratory of neurocognitive genomics, Ufa, Russia Lomonosov Moscow State University, Department of psychology, Moscow, Russia

**Keywords:** nonverbal intelligence, cognitive functions, single nucleotide polymorphism (SNP), association analysis, microglia, inflammatory response, невербальный интеллект, когнитивные функции, однонуклеотидный полиморфный локус, анализ ассоциаций, микроглия, воспалительный ответ

## Abstract

Nonverbal intelligence represents one of the components of brain cognitive functions, which uses visual images and nonverbal approaches for solving required tasks. Interaction between the nervous and immune systems plays a specif ic role in individual differences in brain cognitive functions. Therefore, the genes encoding pro- and antiinflammatory cytokines are prospective candidate genes in the study of nonverbal intelligence. Within the framework of the present study, we conducted the association analysis of six SNPs in the genes that encod proteins involved in inf lammatory response regulation in the central nervous system (CRP rs3093077, IL1А rs1800587, IL1B rs16944, TNF/ LTA rs1041981, rs1800629, and P2RX7 rs2230912), with nonverbal intelligence in mentally healthy young adults aged 18– 25 years without cognitive decline with inclusion of sex, ethnicity and the presence of the “risky” APOE ε4 allele as covariates. Considering an important role of environmental factors in the development of brain cognitive functions in general and nonverbal intelligence in particular, we conducted an analysis of gene-by-environment (G × E) interactions. As a result of a statistical analysis, rs1041981 and rs1800629 in the tumor necrosis factor gene (TNF) were shown to be associated with a phenotypic variance in nonverbal intelligence at the haplotype level (for АА-haplotype: βST = 1.19; p = 0.033; pperm = 0.047) in carriers of the “risky” APOE ε4 allele. Gene-by-environment interaction models, which determined interindividual differences in nonverbal intelligence, have been constructed: sibship size (number of children in a family) and smoking demonstrated a modulating effect on association of the TNF/LTA (rs1041981) (β = 2.08; βST = 0.16; p = 0.001) and P2RX7 (rs2230912) (β = –1.70; βST = –0.10; p = 0.022) gene polymorphisms with nonverbal intelligence. The data obtained indicate that the effect of TNF/LTA on the development of cognitive functions is evident only in the presence of the “unfavorable” APOE ε4 variant and/or certain environmental conditions

## Abstract (ru)

Невербальный интеллект – один из компонентов когнитивных функций мозга, использующий визуальные образы и невербальные методы для решения поставленных задач. Особую роль в развитии индивидуальных различий в уровне когнитивных функций мозга отводят взаимодействию нервной и иммунной систем. Гены, кодирующие про- и противовоспалительные цитокины, могут являться кандидатами при изучении невербального интеллекта. Проведен анализ ассоциаций шести локусов генов, белковые продукты которых принимают участие в регуляции воспалительного ответа в центральной нервной системе: CRP (rs3093077), IL1А (rs1800587), IL1B (rs16944), TNF/LTA (rs1041981, rs1800629), P2RX7 (rs2230912), с уровнем невербального интеллекта у здоровых индивидов без когнитивных нарушений в возрасте 18–25 лет с включением половой, этнической принадлежности и наличия «рискового» аллеля ε4 гена APOE в качестве ковариат. С учетом важной роли средовых факторов, влияющих на формирование когнитивных функций мозга в целом и невербального интеллекта в частности, осуществлен анализ ген-средовых (G×E) взаимодействий. В результате статистического анализа показано, что полиморфные варианты rs1041981 и rs1800629 гена фактора некроза опухоли альфа (TNF) ассоциированы с фенотипическими вариациями в показателе невербального интеллекта на уровне гаплотипов (для гаплотипа АА: βST = 1.19; р = 0.033; pperm = 0.047) в группе носителей «рискового» варианта ε4 гена APOE. Построены модели ген-средовых взаимодействий, детерминирующие межиндивидуальные различия в уровне невербального интеллекта: число детей в семье и табакокурение модулируют ассоциацию вариантов генов TNF/LTA (rs1041981) (β = 2.08; βST = 0.16; p = 0.001) и P2RX7 (rs2230912) (β = –1.70; βST = –0.10; р = 0.022) c уровнем невербального интеллекта. Полученные данные свидетельствуют о том, что эффект гена TNF/LTA на формирование особенностей когнитивной сферы наблюдается только в случае наличия «неблагоприятного» варианта гена АРОЕ и при сочетании определенных социальных факторов.

## Introduction

Understanding the nature of human cognitive development represents one of the relevant issues in the modern-day psychiatric genetics. The possibility to enhance the efficacy of learning at any age directly depends on the knowledge of the mechanisms underlying the development of cognitive functions in the brain and the factors determining this process. Nonverbal intelligence as one of cognitive abilities implies an individual’s ability to use problem-solving strategies and manipulate visual information without using verbal skills (Kuschner, 2013). In turn, verbal intelligence stands for language skills, receptive and expressive speech, vocabulary, and verbal abstract reasoning (Dawson, 2013). The differences in the brain functional architecture are related to the use of verbal and nonverbal skills (Feklicheva et al., 2021).

Nowadays, the study of nonverbal intelligence from a biological point of view is considered the most justified (Vyshedskiy et al., 2017), therefore, individual differences in intellectual development are explained by the effect of a number of physiological factors (anatomical and physiological parameters of the brain, signaling systems, etc.) (Li et al., 2009), which, in turn, are significantly affected by an individual’s genome (Mustafin et al., 2020). Various sociodemographic parameters play an equally important role in the manifestation of individual variance in nonverbal intelligence (Franić et al., 2014).

Inflammatory mediators belong to one of the promising and poorly studied biological systems in relation to nonverbal intelligence. The only immune system cells in the central nervous system (CNS) parenchyma are microglial cells functioning as residential macrophages (Kierdorf, Prinz, 2017). In addition to the barrier function, microglial cells in the mature brain can produce various neurotrophic factors, such as BDNF (brain-derived neurotrophic factor) and GDNF (glial-derived neurotrophic factor) (Parkhurst et al., 2013). Moreover, contemporary studies reported that microglial cells had receptors for neurotransmitters, neuropeptides and neuromodulators (Alekseeva et al., 2019), which indicates a link between microglia and neuronal activity, indicating the prospects for studying the inflammatory system relevant to cognitive functioning of the brain in general and nonverbal intelligence in particular. Accordingly, the genes responsible for regulating the activation and deactivation of microglial cells can mediate the development of nonverbal intelligence.

An important function of microglia is to maintain a balance of pro- and anti-inflammatory processes in the intact brain. Such balance is achieved by the production of anti-inflammatory cytokines by microglia: C-reactive protein (CRP), interleukin 1α (IL1a), interleukin 1β (IL1B), tumor necrosis factor alpha (TNFα). The imbalance in the functioning of microglial cells may cause cytokines accumulation in CNS (Ferro et al., 2021), which, in turn, is one of the reasons for the increased permeability of the blood-brain barrier (BBB). An impaired BBB integration promotes CNS infiltration by leukocytes and neuroinflammation, which may develop into a chronic form and result in abnormal synaptic plasticity of neurons, reduced number of synaptic connections and neurodegenerative processes (Haruwaka et al., 2019). Purinergic receptors are other important participants regulating the inflammatory response. Thus, activation of microglia in the CNS is carried out by purinergic signaling (Franke et al., 2007). According to published data, activation of the purinergic receptor P2X7 initiates innate immunity, thus contributing to an increased level of proinflammatory cytokines (mainly IL-1ß and IL-18) in the CNS, which results in increased inflammation and/or neurons death in various animal species, as well as in humans (Lister et al., 2007).

Another additional genetic risk factor for developing cognitive impairments is the presence of the ε4 variant in the apolipoprotein E (APOE) gene, which, according to literature, is associated with an increased risk of neurodegenerative diseases (Emrani et al., 2020), aging and longevity (Erdman et al., 2016). The APOE protein has three isoforms E2, E3 and E4 encoded by the ε2, ε3, and ε4 alleles, respectively. Multiple data evidence that the APOE reduces inflammation in the CNS in isoform-specific manner: ε2 and ε3 isoforms have anti-inflammatory and protective properties, while ε4 isoform exhibits low anti-inflammatory activity (Lanfranco et al., 2021). In addition, mice lacking the APOE gene demonstrate an enhanced level of proinflammatory cytokines in the CNS (Vitek et al., 2009), which indicates the APOE effect on regulating immune function. Therefore, there is a direct link between the APOE and microglial cells functioning and cytokine production.

Published data indicate the functional significance of rs1800629 (c.-488G>A) and rs1041981 (c.179C>A or Thr60Asn) in the TNF/LTA gene (Hameed et al., 2018), rs2230912 (c.1379A>G or Gln460Arg) in the P2RX7 gene (Winham et al., 2019), rs1800587 (-889C>T) in the promoter region of the IL1А gene (Dominici et al., 2002), rs16944 (-511T>C) in the IL1B gene (Tayel et al., 2018), based on the evidence of modified expression of corresponding genes related to various allelic variants. In addition, a genome-wide association analysis of C-reactive protein levels detected the rs3093077 in the CRP gene in a large cohort of healthy individuals (Naitza et al., 2012).

Considering a functional role of the mentioned SNPs located in the genes involved in the regulation of inflammatory response in the CNS, within the framework of the present study we have performed the association analysis of the CRP (rs3093077), IL1А (rs1800587), IL1B (rs16944), TNF/ LTA (rs1041981, rs18006290), P2RX7 (rs2230912) gene loci with interindividual differences in the level of nonverbal intelligence for the first time. Moreover, a possible modulating effect of the APOE ε4 variant (which is determined based on genotyping of rs7412 and rs429358) and several socio-demographic parameters on the association of inflammatory response genes with nonverbal intelligence has been analysed.

## Materials and methods

The present study involved 1011 individuals (80 % women, mean age 19.79 ± 1.69 years) of different ethnicity (535 Russians, 231 Tatars, 160 Udmurts, and 85 of mixed ethnicity). At the time of participation in the study, all the subjects were students at the universities of Russia and were not registered in the psychiatric database. Informed consent to participate in the study was obtained from all the participants. The design of this study was approved by the Ethics Committee of the Institute of Biochemistry and Genetics UFRC RAS.

The level of nonverbal intelligence was measured using a black-and-white version of the Raven Progressive Matrices (Raven, 2000), which represents the essential and widely used diagnostic instrument for the assessment of examined cognitive construct and is characterized by high validity and reproducibility (Feklicheva et al., 2021). The abovementioned approach includes a representation of figures, which are related to each other by certain dependence. One figure is missing, and a respondent has to choose one missing figure among 6–8 figures presented. The respondent has to establish a pattern connecting the figures and to choose a missing figure among the presented ones. The test consists of 60 tables (5 series). The complexity of tasks enhances with an increase in the series and the task number in each series of tables.

To estimate a modulating effect of several socio-demographic parameters, which had been previously reported to influence cognitive abilities, the respondents were asked to present information regarding their ethnicity up to three generations, birth order and the sibship size, smoking status, rearing style, the presence of mental illness in close relatives, knowledge of their native language (Bashkir, Tatar, Udmurt, etc.). Information about the rearing style included such questions on child-parent relationships as the episodes of childhood maltreatment, rearing in a complete/incomplete family, family income, and maternal age at delivery of the respondent.

DNA samples isolated from the venous blood by phenolchloroform extraction were used in the present study. A genotyping of the IL1A (rs1800587), CRP (rs3093077), TNFα (rs1041981, rs1800629), P2RX7 (rs2230912), and APOE (rs7412, rs429358) gene SNPs was performed by real-time PCR with a fluorescent detection using the KASP method. Detection was carried out on the CFX96 DNA Analyzer (Bio-Rad, USA) with the endpoint fluorescence analysis. The genotypes in the APOE gene were grouped based on the presence of ε2, ε3, ε4 alleles

The estimate of allele and genotype frequencies distribution was conducted using the Hardy–Weinberg equilibrium test. To verify the correspondence of scores distribution obtained via the Raven’s progressive matrices to the Gaussian distribution, we performed the Shapiro–Wilk W-test. To assess the main effect of gene polymorphisms together with gene-environment interactions (G×E) in individual variance in nonverbal intelligence, a series of linear regression analyses was carried out. Genotypes and social parameters were used as independent factors in the G×E analysis, while the level of nonverbal intelligence was used as a dependent variable. Statistical analysis included the verification of several linear regression models (additive, dominant, recessive); sex, ethnicity and the “risky” APOE ε4 allele were included as covariates. A correction for multiple comparisons was carried out using the FDR (false discovery rate) procedure or permutation (10,000) in the case of haplotype analysis. Statistical analysis was performed using the programs PLINK v.1.09, R, SPSS Statistics 23.0. Visualization was conducted in R v.4.1.2.

## Results

Within the present study we analyzed the effect of eight SNPs in six genes: CRP (rs3093077), IL1А (rs1800587), IL1B (rs16944), TNF/LTA (rs1041981, rs1800629), P2RX7 (rs2230912), APOE (rs7412, rs429358), which are involved in the regulation of inflammatory system, on individual differences in nonverbal intelligence in mentally healthy individuals. According to the Shapiro–Wilk W-test, nonverbal intelligence scores were congruent to the normal distribution ( p > 0.05). A distribution of allele and genotype frequencies of all examined loci corresponded to the Hardy–Weinberg equilibrium ( p > 0.05). The analysis of allele and genotype frequencies distribution demonstrated the absence of statistically significant differences between various ethnic groups ( p > 0.05); therefore, a subsequent statistical analysis was conducted in the total group with previously reported requirement to include sex and ethnicity as covariates, as well as in men and women separately

**Fig. 1.**
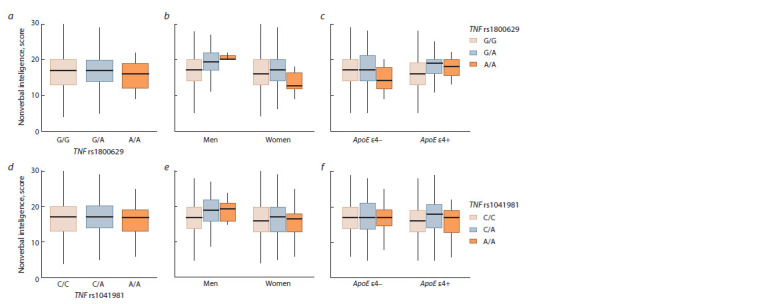
Mean values of nonverbal intelligence depending on the genotypes in the TNF rs1800629 and rs1041981 gene polymorphisms in the total sample (a, d ), in the groups differing by sex (b, e) and the presence/absence of the APOE ε4 variant (c, f ).

Statistical analysis of associations of eight SNPs in six genes involved in the regulation of inflammatory response with nonverbal intelligence, which was conducted via linear regression, revealed the effect of TNF (rs1800629) (for additive model: βST = 0.15; p = 0.022; pFDR = 0.137; for dominant model: βST = 0.16; p = 0.019; pFDR = 0.059) and rs1041981 (for dominant model: βST = 0.16; p = 0.019; pFDR = 0.059) on individual differences in nonverbal intelligence among carriers of the “risky” APOE ε4 variant (see the Table, Fig. 1, c, f ). However, this association became only a trend after correction for multiple comparisons. In particular, the carriers of rs1800629 and rs1041981 minor A-alleles demonstrated an increased nonverbal intelligence compared to individuals bearing G/G and C/C-genotypes (respectively) at the trend level, which was evident only under genetically determined diminished anti-inflammatory activity (AРОЕ ε4 variant). In men, the association of TNF gene loci appeared to become insignificant after FDR-correction (see the Table, Fig. 1, b, e).

**Table 1.**
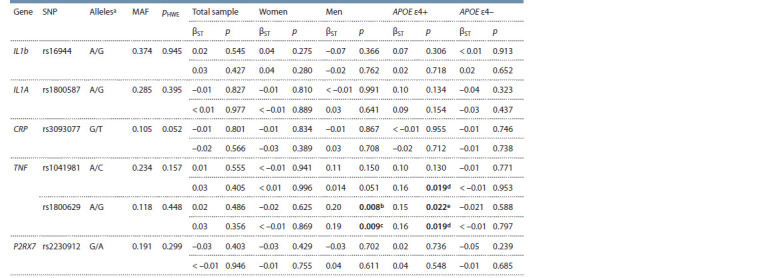
Examined SNPs, the Hardy–Weinberg equilibrium test and the results of linear regression analysis of association of the genes with a nonverbal intelligence in the total sample and in subgroups Notе. pHWE – p-value for the Hardy–Weinberg equilibrium test; MAF – minor allele frequency; βST – standardized regression coeff icient; p – р-value. The upper row indicates the results obtained from an additive model, the lower row – from a dominant model. Statistically signif icant values are shown in bold. a Minor/major alleles; b pFDR = 0.051; c pFDR = 0.058; d pFDR = 0.059; e pFDR = 0.137.

The linkage disequilibrium analysis conducted between the TNF (rs1800629 and rs1041981) succeeded to report a linkage (D′ = 0.741, r2 = 0.235), therefore, a haplotypic analysis was performed. Haplotype frequencies in the TNF gene (based on rs1041981, rs1800629) were the following: AA – 0.094, CA – 0.023, AG – 0.143, CG – 0.740. As a result of haplotypic analysis we detected the association of the TNF*АА haplotype (rs1041981, rs1800629) (βST = 1.19; p = 0.033; pperm = 0.047) with an enhanced level of nonverbal intelligence in individuals without cognitive decline, which remained statistically significant after correction for multiple comparisons.

**Fig. 2.**
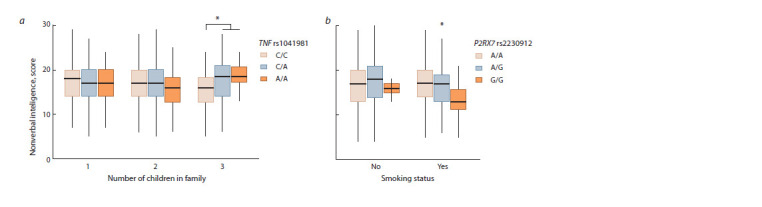
The results of gene-by-environment interaction analysis demonstrating a modulating effect (a) of the sibship size on the association of the TNF (rs1041981) and (b) smoking on the association of the P2RX7 (rs2230912) with nonverbal intelligence. Statistically significant differences in nonverbal intelligence between the groups are marked with brackets. *pFDR < 0.05.

In the present study we also conducted the analysis of gene-by-environment (G×E) interactions estimating the effect of 14 socio-demographic parameters. As a result of G×E interactions we observed that sibship size significantly affected the association of the rs1041981 in the TNF gene (β = 2.08; βST = 0.16; p = 0.001). Thus, it was revealed that carriers of the rs1041981 А-allele who were reared in the families with three and more children demonstrated a significantly higher level of nonverbal intelligence compared to those with the rs1041981 C/C-genotype (Fig. 2, a). Moreover, we observed that smoking had a modulating effect on the association of the P2RX7 rs2230912 (β = –1.70; βST = –0.10; р = 0.022) with individual differences in nonverbal intelligence. In particular, we observed a dose-dependent effect of P2RX7 rs2230912 minor G-allele on a decreased level of nonverbal intelligence among smoking individuals (see Fig. 2, b). In the present study, we failed to observe associations of the IL1В (rs16944), IL1A (rs1800587), CRP (rs3093077) gene polymorphisms with individual differences in nonverbal intelligence.

## Discussion

The inflammatory response system plays an important role in the development and normal functioning of cognitive abilities (Sartori et al., 2012; Fard, Stough, 2019). Within the framework of the present study, an attempt was made to identify evidence of the involvement of genes encoding inflammatory system proteins in the manifestation of individual differences in nonverbal intelligence. The results of our study identified the effect of tumor necrosis factor alpha (TNF) and purinergic receptor P2X7 (P2RX7) genes and social parameters such as smoking and sibship size in childhood on the development of cognitive abilities. Previously, we had also identified a modulating effect of sibship size on manifestation of cognitive abilities (Kazantseva et al., 2016). The results obtained can provide valuable information for determining genetic mechanisms underlying the development of cognitive functions in general and nonverbal intelligence in particular.

To date, scarce research has been devoted to the study of the inflammatory response system related to normal cognitive functioning in the brain. There may be several explanations for this. Cognitive functions represent a complex personality construct, the development of which is based on a large number of both biologically determined and environmental factors (Xu et al., 2015; Wang et al., 2019). In this regard, genetic data related to this cognitive phenotype are accumulating gradually, while the majority of studies are devoted to the examination of more obvious biological systems that can directly affect the transmission of information between the neurons, neurogenesis, differentiation of neurons, and others (Kazantseva et al., 2020, 2021). The second reason may be attributed to the assumption that the brain as an organ is completely isolated from immune processes. However, to date, more findings on the cellular components of innate and acquired immunity represented in the brain have been published (Filiano et al., 2015; Morimoto, Nakajima, 2019).

The inflammatory response refers to nonspecific innate immunity that occurs as a response to pathogen penetration. Scientific publications indicate that the inflammatory process in the brain is primarily associated with microglia functioning (Li, Barres, 2018), which represent a large population of immune cells in the central nervous system (Ginhoux et al., 2010). One of the main functions of microglia is to maintain the balance of inflammatory and anti-inflammatory processes in the intact brain (Li, Barres, 2018). The imbalance in these processes can transform into a pathological process, which initiates endogenous neuroinflammation (Wake et al., 2011) and damages neuronal integrity. In turn, the latter may be caused by the factors responsible for the activation of microglia and affect cognitive processes in the brain. This observation may partly explain the associations of SNPs in the gene encoding tumor necrosis factor alpha (TNF) with variations in nonverbal intelligence determined in the present study. Within the present study we demonstrated the association of minor alleles of TNF rs1800629 and rs1041981 polymorphisms (at the haplotype level) with a higher level of nonverbal intelligence in mentally healthy individuals. The TNFα protein is one of the pro-inflammatory cytokines, which plays an important role in the initiation and regulation of the cytokine cascade during the inflammatory reaction (Makhatadze, 1998). According to literature, TNFα deficiency results in uncontrolled inflammatory response, which, in turn, can cause chronic course of the inflammatory process and negatively affect the integrity of neurons (Raffaele et al., 2020).

The examined rs1800629 (c.-488G>A) in the TNF gene and rs1041981 (c.179C>A or Thr60Asn) in the LTA gene are functionally significant, and minor alleles are associated with an increased expression of the TNF/LTA genes (Hameed et al., 2018), which indicates that our data are consistent with previous research. Based on the data obtained, it can be assumed that an enhanced expression of the TNF gene is protective and contributes to more controlled inflammatory process in the brain, which positively affects human cognitive functioning.

It should be noted that a positive effect of minor alleles in the TNF gene on improving cognitive performance was observed only in the presence of the “unfavorable” ε4 allele in the APOE gene. Together with the involvement of the APOE protein in cholesterol metabolism, it also demonstrates an immunomodulatory effect and the evidence indicating the role of the APOE in developing neurodegenerative diseases increasingly appear in the literature. To date, it is known that APOE can alter the CNS response to acute and chronic damage, thus actively regulating microglia activation and deactivation (Fitz et al., 2021).

The association of polymorphic variants in the TNF gene with nonverbal intelligence observed in the present study in carriers of the APOE “risky” ε4 variant can be explained by a close link of these proteins in the human body. In the study conducted by D.T. Laskowitz et al. (1997) it was shown that APOE was able to suppress the secretion of TNFα by glial cells, while TNFα deficiency in the CNS resulted in imbalanced inflammatory and anti-inflammatory processes in the intact brain. Thus, a favorable effect of the presence of minor alleles in the TNF gene (at the haplotype level) on cognitive abilities may be attributed to APOE-related changes in TNFα secretion and, hence, to a certain level of neuroinflammation

The results of gene-environment studies are interesting. Thus, it was shown that the number of children in a family (sibship size) had a modulating effect on the association of the TNF rs1800629 with variations in nonverbal intelligence. In the literature, there are multiple contradictory findings concerning the role of the “intellectual climate” in the family in intelligence level. The results of the majority of studies indicate that younger children are less successful in learning and have lower scores on cognitive tests compared with their older siblings (Kanazawa, 2012). Such observations are explained by the fact that one child in the family receives more parental attention and time resources, while the appearance of each subsequent child is accompanied by insufficient parental time and resources. Nevertheless, such patterns are more relevant to verbal intelligence and may be extended to nonverbal one (Blake, 2020).

In the present study, no differences in cognitive abilities depending on the sibship size were observed. Nevertheless, the association of a higher level of nonverbal intelligence with TNF rs1041981 minor A-allele was observed only among individuals who were reared in a large family, while in groups of individuals with a different sibship size no TNFdependent association with cognitive indicators was obtained. From another point of view, nonverbal intelligence positively correlates with family size, since children in large families demonstrate a better ability to understand nonverbal signals due to a decrease in verbal contacts (Morand, 1999). Therefore, genetically determined pro-inflammatory response of the organism associated with the expression of the TNF gene plays a significant role in the development of nonverbal intelligence in the case of rearing in a large family, which promotes the development of nonverbal processes (Morand, 1999). The data obtained by our group indicate a favorable effect of the TNF rs1041981 minor A-allele, which is associated with more controlled inflammatory process in the brain, on nonverbal intelligence, which manifests only under the conditions of limited verbal parental resources (i. e., a large family).

The second statistically significant result of gene-by-environment analysis carried out in the present study evidences a modulating effect of smoking on the association of the P2RX7 rs2230912 with nonverbal intelligence. Namely, carriers of the rs2230912 G-allele in the P2RX7 gene demonstrated a decreased level of nonverbal intelligence in smokers compared with carriers of other genotypes. The P2RX7 receptor belongs to the purinergic signaling system, which regulates interaction of neurons and the functioning of glial cells, primarily, microglia (Lister et al., 2007).

According to literature, A to G transition in the P2RX7 rs2230912 is accompanied by glutamate replacement with arginine at position 460, which is expressed in modified signal transmission by the translated P2RX7 protein (Winham et al., 2019). This receptor is involved in the secretion and degradation of extracellular ATP belonging to inflammationinducing molecules. An impaired ATP metabolism results in enhanced concentration of this molecule in the intercellular space, which can promote a chronic inflammatory process in the CNS and negatively affect the integrity of neurons (Shevela et al., 2020). Another reason of abnormal ATP metabolism in the organism is cigarette smoke. One of the mechanisms of the effect of cigarette smoke on ATP metabolism may be attributed to its ability to modify the expression of the TSPO gene encoding the translocator protein, which is increased in the outer mitochondrial membrane responsible for ATP synthesis (Zeineh et al., 2019). In addition, recent studies linked cognitive impairment with nicotine addiction and the number of cigarettes smoked per day. According to large-scale longitudinal studies involving individuals with nicotine smoking, a decreased working memory volume and the ability to solve problems was revealed (Vermeulen et al., 2018).

The examined SNP (G-allele) and an increased expression of the P2RX7 protein were previously associated with a risk for developing affective and depressive disorders (Winham et al., 2019), which is partially consistent with our results on a lower level of cognitive functioning in carriers of the rs2230912 G-allele, which manifests only under the conditions of enhanced neuroinflammatory reaction (such as smoking). Based on the abovementioned data, it can be assumed that a reduced level of nonverbal intelligence may be related to the changes in ATP metabolism and associated neuroinflammatory process.

## Conclusion

The present study has several limitations, since the results were obtained using an average sample size. Another limitation is the small number of examined gene polymorphisms, which makes our conclusions on the involvement of the inflammatory system genes incomplete. It should also be noted that within the framework of this study, no genetic correlation was assessed between the level of nonverbal and verbal intelligence, as well as other cognitive abilities, which does not allow us to make an unambiguous conclusion on the specificity of demonstrated genetic associations specifically for nonverbal intelligence. Nevertheless, the results obtained in the present study make a significant foundation and set a direction for the study of genetically determined factors underlying the studied cognitive ability

This research also has several strengths: for the first time, the association analysis of the genes involved in the regulation of the inflammatory response with nonverbal intelligence was carried out. In addition, this study also includes the analysis of gene-by-environment interactions, which helps to understand the biological nature of nonverbal intelligence and the role of the immune system in the manifestation of interindividual differences in this cognitive construct in mentally healthy individuals.

## Conflict of interest

The authors declare no conflict of interest.
